# Synthesis, characterization, computational, antioxidant and fluorescence properties of novel 1,3,5-trimesic hydrazones derivatives

**DOI:** 10.1016/j.heliyon.2021.e08074

**Published:** 2021-09-25

**Authors:** Ibrahim Mhaidat, Fadel Alwedian, Taher Ababneh, Ayman Shdefat, Hasan Tashtoush

**Affiliations:** Department of Chemistry, Faculty of Science, Yarmouk University, Irbid, 1163, Jordan

**Keywords:** Trimesic acid, Hydrazones, Antioxidant, Spectrophotometric, Fluorescence, DFT

## Abstract

New photophysical and antioxidant materials of trimesic trihydrazide derivatives were synthesized by one-pot stage of trimesic trihydrazide and different aromatic aldehydes. All compounds were characterized by spectroscopic techniques (NMR, MS, and IR) and elemental analysis. The absorption and emission spectral characteristics of hydrazone derivatives were investigated. The absorption maxima showed red shift relative to the starting compound. While the emission maxima showed clear dependent on the type of substituents. The electron donating and electron withdrawing showed red and blue shifts relative to the starting compound, respectively. The compounds’ effectiveness as antioxidant was estimated by DPPH radical scavenging and ABTS radical cation assays in vitro which indicated that the derivatives could be used as potential antioxidants. In addition, compounds 3g, and 3i showed strong antioxidant activities according to the DPPH assay and compounds 3c and 3m exhibited good antioxidant activities in ABTS assay. Antimicrobial activity of the derivatives was estimated using a micro-broth dilution method. Furthermore, molecular geometries of all prepared derivatives were fully optimized using density functional theory (DFT) calculations at the 6-31G(d)/B3LYP level of theory.

## 1 Introduction

Hydrazones are a special class of Schiff bases with a general structure R_1_R_2_C = NNH_2_ [1, 2]. Their structures contain both electron-donating and electron-withdrawing substituents and are basically related to ketones and aldehydes [3]. The combination of hydrazones with other functional groups leads to a compound with uniquely physical and chemical characteristics which are greatly affecting their biological and pharmacological properties.

Hydrazones are commonly used as biologically active reagents to treat several diseases including anti-microbial [4] anti-tumor [5, 6], tuberculosis [6], leprosy and mental disorder [7]. In addition, they were used as herbicides, insecticides, nematicides, rodenticides and plant growth regulators [8]. Antioxidants are responsible for the defense mechanisms of organisms against pathologies associated with the attack of free radicals that can be produced by the oxidation reactions, which in turn, can initiate chain reactions. It is well established that free radical chain reaction in the cell may cause its damage or death. Antioxidants terminate these chain reactions by removing free radical intermediates and inhibit other oxidative reactions [9, 10, 11, 12]. Experimentally, arylhydrazone compounds can function as free-radical scavengers [13, 14] and as antioxidant agents [15, 16, 17].

The aryl substitution pattern of hydrazones produced a unique structure of arylhydrazone. This structure is greatly affecting both luminescence efficiency and photophysical properties of hydrazones [18]. A wide range of triazine and hydrazone-containing molecules has been studied using DFT methods. Computational analysis is carried out to confirm the experimental findings and to investigate further structural features [19, 20, 21].

The present work aims to synthesis and characterization of a new substituted arylhydrazones derived from trimesic acid. Their molecular structures were identified using elemental analysis, ^1^H-NMR, ^13^C-NMR, FT-IR, and UV-vis spectroscopy, and further confirmed by DFT computational study. The antioxidant and biological activities of the synthesized molecules were also investigated.

## 2 Results and discussion

### 2.1 Synthesis and characterization of trimesic hydrazons **(3a-3m)**

**Figure 1:**
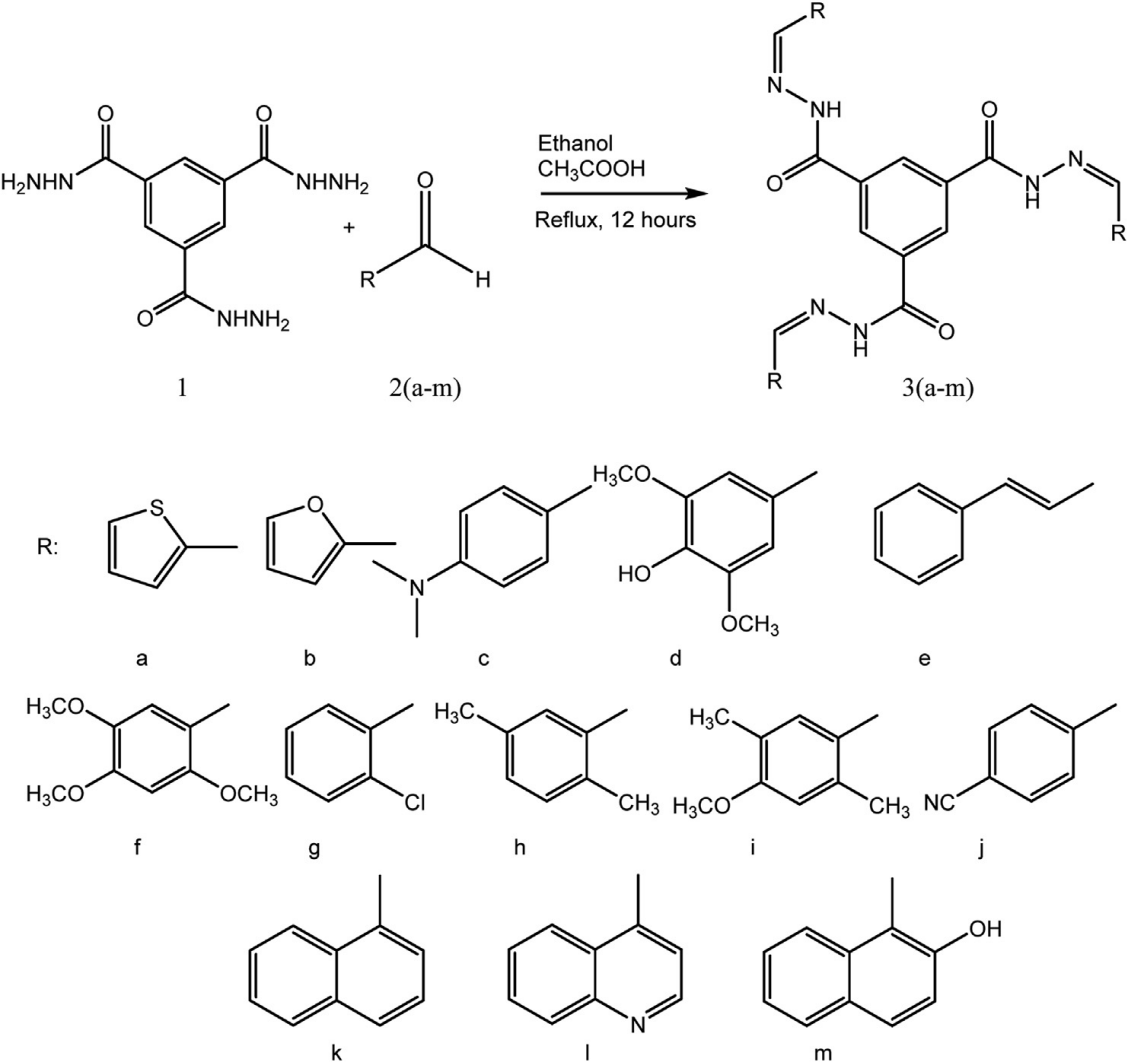
The synthetic route for trimesic hydrazones (3a-3m).

The synthetic routes of the trimesic hydrazone (**3a**-**3m**) are summarized in Figure 1. Hydrazone compounds were synthesized as specified by two reactions: methyl trimesic ester reacts with hydrazine hydrates to yield trimesic trihydrazide followed by a condensation reaction with aromatic aldehydes. The process provided excellent yields (85–95%). The IUPAC names, yields, melting points, elemental analysis of (C, H and N), IR and NMR are summarized in the experimental part. The Infrared spectra of the synthesized compounds reveal the bands at 3187–3271, 1649–1671, 1512–1591 cm^−1^ which are corresponding to the stretching frequencies of NH, C=O and C=N functional groups of trimesic hydrazine compounds, respectively. In addition to that, other characteristic bands at 3394 and 3441 cm^−1^ assigned to ν (OH) for **3d** and **3m**, respectively, and a band at 2360 cm^−1^ due to ν (C≡N) for **3j**. The ^1^H-NMR spectra of **3a**-**3m** compounds show a singlet peak at 8.3–8.9 ppm of azomethine protons (–CH = N-) and a singlet band at 11.85–12.75 ppm of imino protons (-NH). Moreover, the aromatic protons appear on the aromatic regions. Similarly, the ^13^C-NMR spectra for all compounds show peaks at 161.0 to 163.0 and 142.0–146.0 ppm for carbonyl (C=O) and imine (C=N) groups, respectively. Moreover, the spectra of **3d**, **3f**, and **3i** show peaks in the range of 55.0–57.0 ppm due to the presence of methoxy group; **3c** exhibits a peak at 39.7 ppm for alkyl amine; **3h** and **3i** display several peaks in the range of 16.0–24.0 ppm due to the presence of alkyl groups. The aromatic carbons appeared in the expected aromatic regions.

### 2.2 Biological activity

#### 2.2.1 Antimicrobial properties

All synthesized compounds were screened as a potential antimicrobial agent. Their antimicrobial activities were tested in vitro against Gram positive bacteria and Gram negative bacteria, including (*Eschereshia coli; Klebsiella pneumonia; Proteus mirabilis; Salmonella enteritidis, Pseudomonas aeruginosa; Staphylococcus aureus; Enterococcus faecalis; Bacillus cereus*). Unfortunately, all these compounds were inactive against all tested bacteria used in this study. The range of concentrations was in range from 500 to 0.5 μg/mL of the respective compounds under investigation with positive and negative control well. Amoxicillin and DMSO were used as positive and negative controls. The MIC of all tested chemicals were above 500 μg/mL and considered inactive.

#### 2.2.2 Scavenging radical activity on 2,2-diphenyl-1-picrylhydrazyl (DPPH) radical

**Figure 2:**
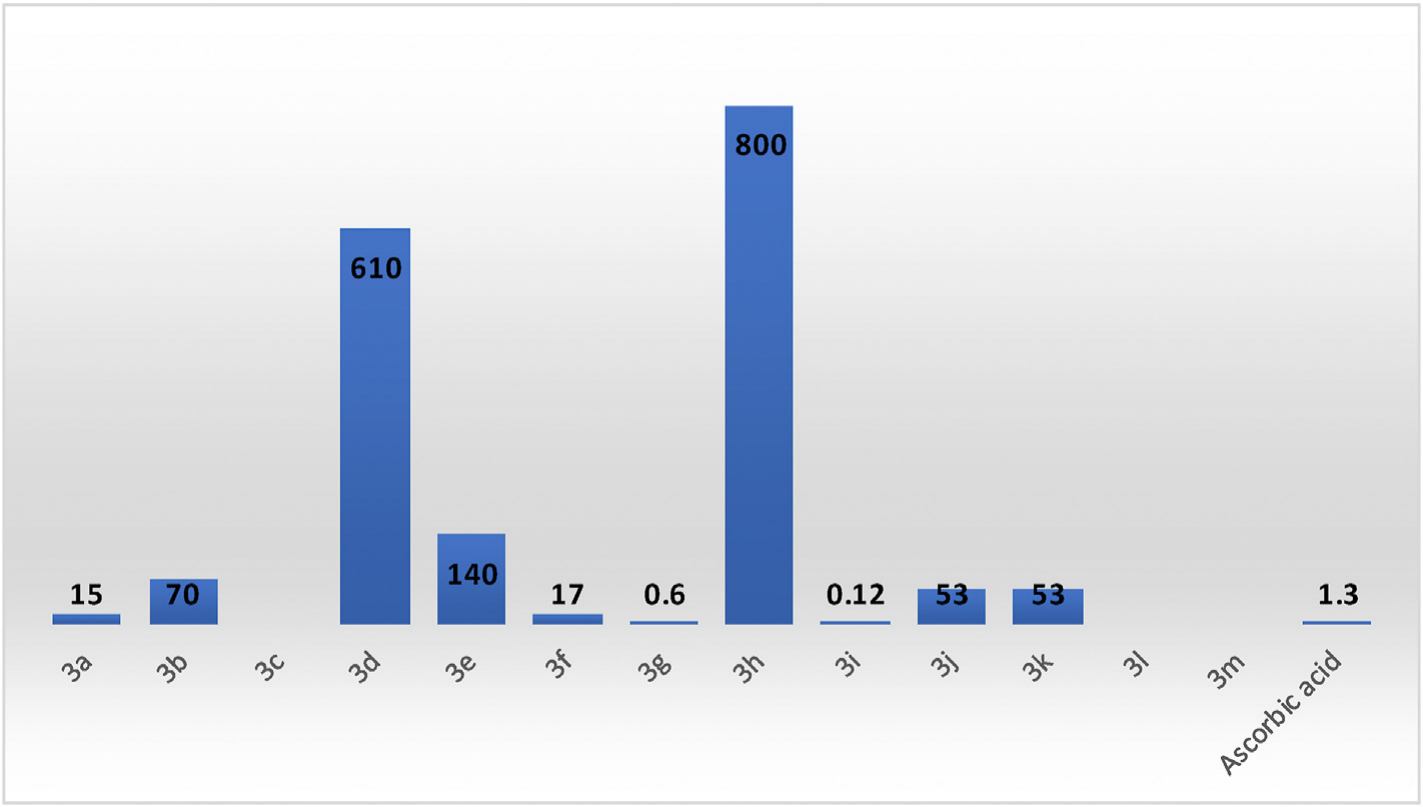
IC_50_ (μg/mL) values (concentration needed for reducing DPPH absorption by 50% at 517 nm) of compounds 3a,3b,3e, 3f, 3g, 3i, 3j, 3k, and ascorbic acid. No data for 3c, 3l and 3m in DPPH assay were presented due to lack of activity.

Compound 3i (with a methoxy and two methyl groups on phenyl) and compound 3g (with a Cl group in the ortho-position of phenyl) showed the best inhibitory activities with 0.12 and 0.6 μg/mL IC_50_ values, respectively. The 3i and 3g had higher antioxidant activities than the ascorbic acid with 1.3 μg/mL IC_50_ value. Compounds 3a with the thiophene ring and 3f with the three methoxy groups in positions 2,4, and 5 of phenyl showed strong antioxidant activities with the 14.0 and 17.0 μg/mL IC_50_ values, respectively; however, these compounds showed lower activities relative to the ascorbic acid. Compounds 3b, 3j, and 3k exhibited moderately antioxidant activities with 70, 53, and 53 μg/mL IC_50_ values, respectively. Compounds 3d, 3e, and 3h showed weak antioxidant activities with 610, 140 and 800 mg/mL IC_50_ values, respectively. While compounds 3c, 3l and 3m did not show any antioxidant properties, see Table 1 and Figure 2.

**Table 1:** Antioxidant activity results of synthesized compounds 3a-3m.

Compound	IC_50_ (μg/mL) DPPH	IC_50_ (μg/mL) ABTS
**3a**	15	28
**3b**	70	26
**3c**	ΝΑ	13
**3d**	610	17
**3e**	140	27
**3f**	17	23
**3g**	0.6	27
**3h**	800	41
**3i**	0.12	29
**3j**	53	19
**3k**	53	35
**3l**	ΝΑ	28
**3m**	ΝΑ	14
Ascorbic acid	1.3	2

#### 2.2.3 Scavenging radical activity on 2,2′-azinobis(3-ethylbenzthiazoline-6-sulfonic acid) (ABTS^•+^) radical cation

**Figure 3:**
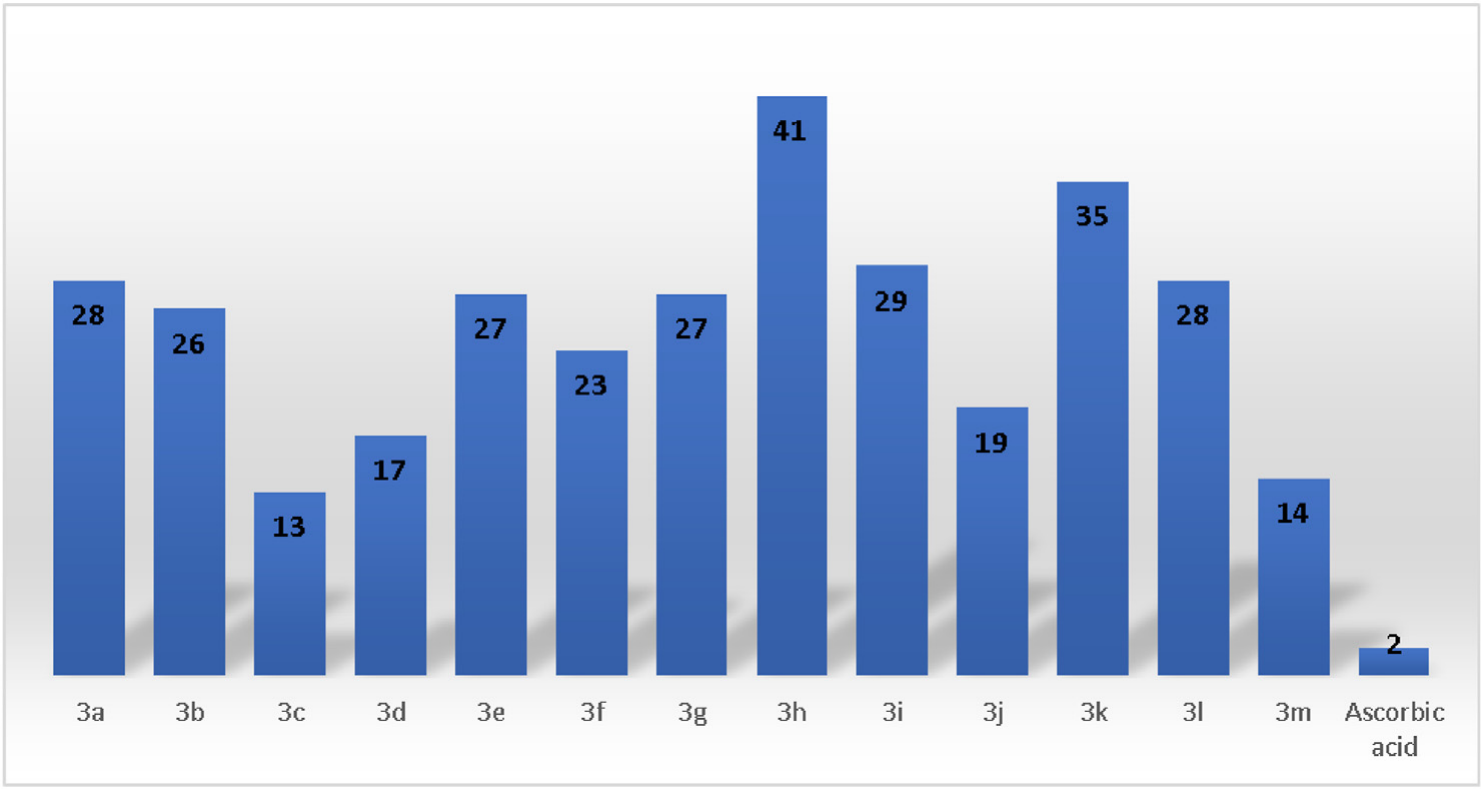
IC_50_ (μg/mL) values (concentration needed for reducing **ABTS**^**•+**^ absorption by 50% at 734 nm) of compounds 3a-3m ascorbic acid.

Compounds 3a-3m established strong to moderately antioxidant activity with 13–41 μg/mL IC_50_ values in ABTS assay. Compound 3c with a N,N-dimethylamino group in the para-position of phenyl and 3m with a hydroxy group of naphthyl had the most antioxidant behavior among the tested compounds in ABTS assay with the 13 and 14 μg/mL IC_50_ values, respectively. Compounds 3d, 3j, 3f, 3b,3e, 3g, 3a, 2l, and 3i indicated good **ABTS**^**•+**^cation radical scavenging activities with 17, 19, 23, 26, 27, 27, 28, 28, and 29 μg/mL IC_50_ values, respectively. On the other hand, compounds 3k and 3h showed moderately activities with 35 and 41 μg/mL IC_50_ values, respectively. All the compounds exhibited lower antioxidant activities compared to the ascorbic acid, see Table 1 and Figure 3.

The antioxidant results did not provide any direct relationship between the position and the type of substituents on aromatic ring and the antioxidant activity of all tested compounds, some authors reported similar finding [22, 23, 24, 25, 26, 27].

### 2.3 The spectrophotometric and fluorometric measurements

**Figure 4:**
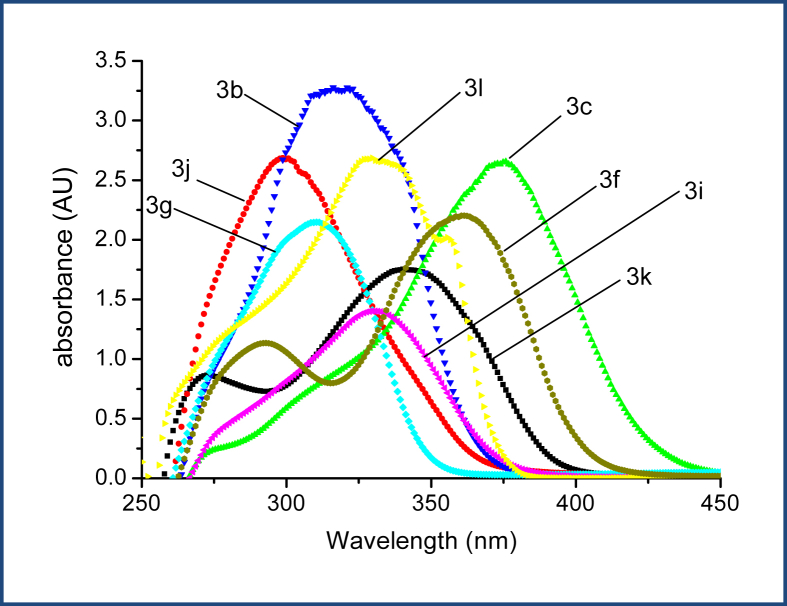
The UV–vis spectra for the hydrazones (**3b**, **3c**, **3f**, **3g**, **3i**, **3j**, **3k** and **3l**) in DMSO at a room temperature.

The Ultraviolet–visible spectra of the compounds **3a**-**3m** were measured in DMSO. Figure 4 shows the UV spectra of selected compounds (**3b**, **3c**, **3f**, **3g**, **3i**, **3j**, **3k** and **3l**), with the corresponding maximum wavelengths of new compounds **3a**-**3m** recorded in Table 2. The UV–vis spectra of all compounds exhibited λ_max_ between 295–366 nm, which is attributed to π to π∗ transitions. Also, it was observed that compounds with electron-donating substituents such as amino group (-N(CH_3_)_2_, exhibited longer maximum wavelengths. On the other hand, compounds with electron-withdrawing substituents had shorter maximum wavelengths.

**Table 2:** The maximum wavelengths of absorption and emission of compounds 3a-3m.

Compound	λ_abs_ (nm)	λ_em_ (nm)	Compound	λ_abs_ (nm)	λ_em_ (nm)
**3a**	325	480	**3h**	310	430
**3b**	320	400	**3i**	330	465
**3c**	370	516	**3j**	295	404
**3d**	330	450	**3k**	340	430
**3e**	335	460	**3l**	330	405
**3f**	360	422	**3m**	335	440
**3g**	310	402			

**Figure 5:**
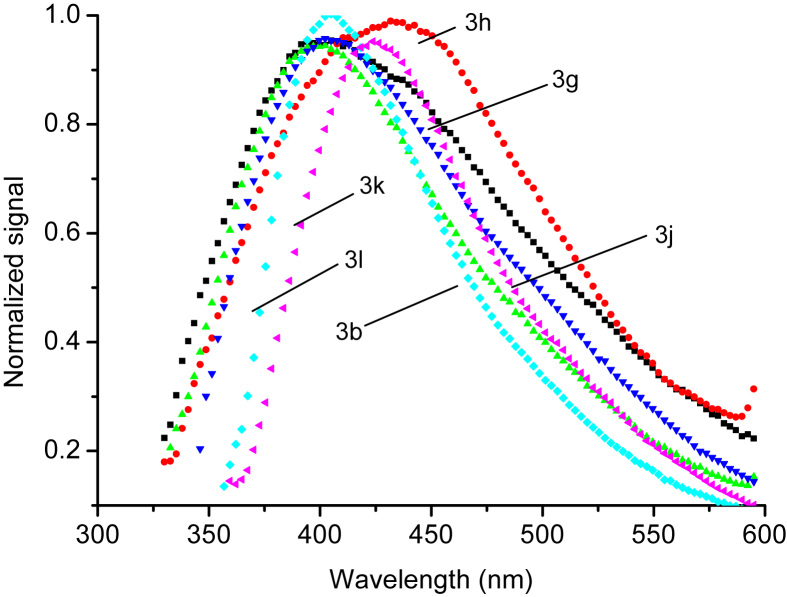
The normalized fluorescence spectra of (3b,3g, 3h, 3j, 3k, and 3l) in DMSO at room temperature.

**Figure 6:**
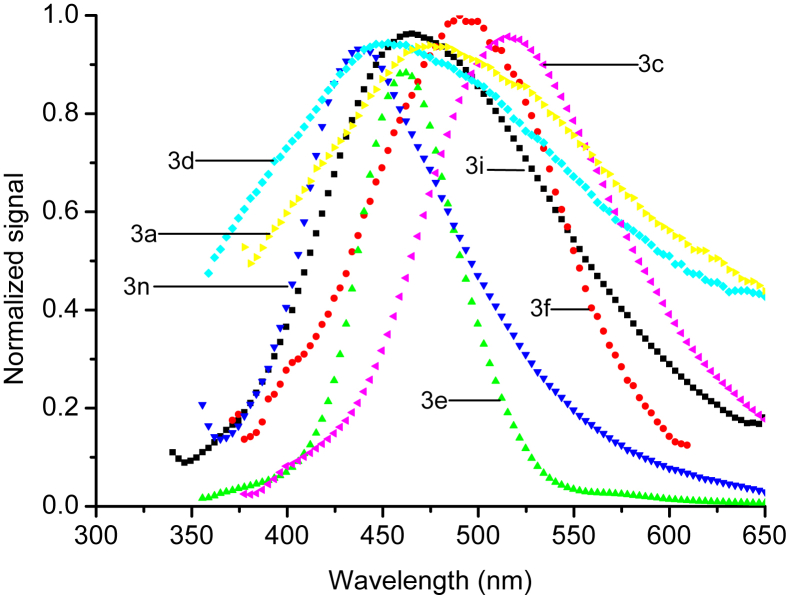
The normalized fluorescence spectra of (3a, 3c, 3d, 3e, 3i, 3f, and 3m) in DMSO at room temperature.

The normalized fluorescence spectra of all compounds were recorded under similar conditions using DMSO as solvent, see Figures 5 and 6. The maximum emission wavelengths for all compounds are listed in Table 2. The effect of substituents on the emission maxima are divided into three groups. The aromatic ring substituent on compounds 3h and 3k show no significant influence on the fluorescence properties. Compounds 3a, 3c, 3d, 3e, and 3m show red shifts relative to the starting compound. This may be correlated with influence of electron-donating groups. On the other hand, compounds 3b, 3f, 3g, 3j and 3l showed blue shifts by about 25 nm, which may be correlated to presence of electron-withdrawing groups. These results were in good agreement with other published studies [18, 23].

### 2.4 Computational study

**Figure 7:**
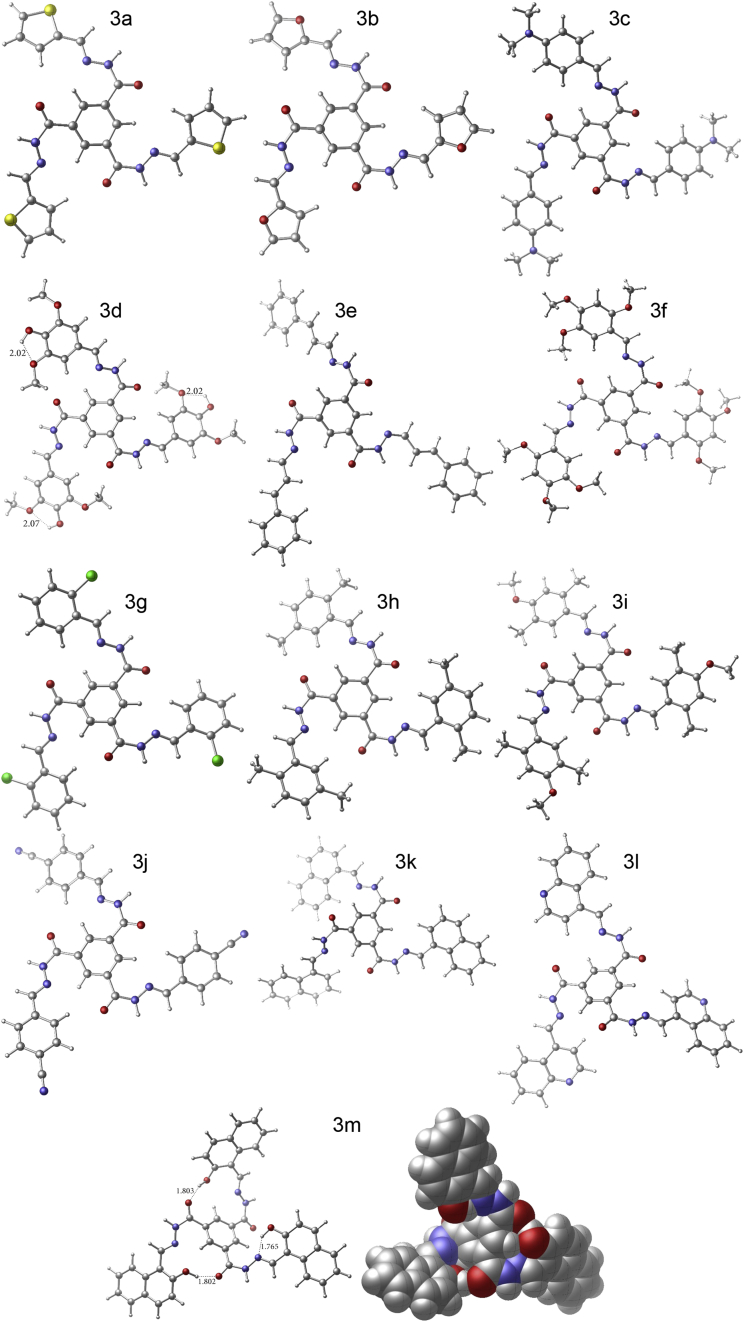
The optimized ground-state geometries of **3a**-**3 m** at the 6-31G(d)/B3LYP level of theory (structure of 3m is also depicted in space-filling view showing propeller shape).

We have undertaken an intensive theoretical investigation in order to find the most-stable minimal-energy structure for each derivative. The methodology considers all initial molecular orientations at the semiempirical/PM6 quantum chemical level by running a conformer distribution calculation. After exploring all possible conformers with such flexible molecules, the search is narrowed down and a higher DFT level of theory is applied to finally obtain the optimized structures. The absence of imaginary frequencies in the vibrational mode calculation confirmed true minima on the potential energy surface for each derivative. The optimized ground-state geometries of derivatives (**3a**-**3m**) at the B3LYP/6-31G(d) level of theory are depicted in Figure 7. All structures feature trimeric propeller-shaped arrangements around a central moiety of 1,3,5-trisubstituted benzene. To get a better insight into the extent of spatial twist exhibited by each of the three aryl moieties in reference to the central trisubstituted arene backbone, the angles between the central benzene plane and cyclic aryl substituents were calculated. For each tripodal molecule, the three dihedral angles and their average are listed in Table 3. Average values ranged from 18.18°to 53.39°. While compounds **3a**-**3j** (all monocyclic aryl side groups) show relatively small variation in average dihedral angles among them, compounds **3k**, **3l** and **3m** (all bicyclic aryl side groups) have noticeably higher values 40.83°, 38.98° and 53.39°, respectively. This reflects the direct impact of structure on orientation of substituents. The closest compound to a planar geometry is **3b** with an average dihedral angle of 18.18°. on the other hand, compound **3m** has the largest average dihedral angle 53.39°, which could be attributed to steric requirements as well as being the only compound with the presence of H-bonding between adjacent substituents (H-bond lengths (Å) are shown in Figure 6. Notice that compound **3d** also features H-bonding, but it is within the same substituent, between a phenolic hydrogen and methoxy oxygen (phO–H···O–Me). The results are comparable to trimer structures reported elsewhere [28]. This class of molecules containing aromatic dimer and trimer side groups is of great interest in biological systems. For instance, aromatic trimer-containing molecules are present in several proteins and are considered the building blocks for higher-order clusters [29, 30]. Therefore, it may be of interest for future research to investigate this series of compounds and expand it to other aromatic side groups found in proteins with the potential of biological activity and as promising building units for new assemblies.

**Table 3:** Dihedral angles (°) between central benzene ring plane and aryl substituents of **3a**-**3m**.

Compound	Dihedral angles			Average
**3a**	18.34	12.56	24.90	18.60
**3b**	16.62	14.20	23.71	18.18
**3c**	16.27	25.19	19.93	20.46
**3d**	31.31	27.77	22.06	27.05
**3e**	30.70	28.86	35.27	31.61
**3f**	26.37	27.63	15.79	23.26
**3g**	26.45	30.96	20.00	25.80
**3h**	20.81	24.84	25.63	23.76
**3i**	23.43	18.42	23.95	21.93
**3j**	19.30	22.63	27.95	23.29
**3k**	42.25	36.75	43.50	40.83
**3l**	38.00	39.13	39.82	38.98
**3m**	44.68	59.86	55.64	53.39

## 3 Materials and methods

Solvents and chemical compounds such as methanol, ethanol, DMF, DMSO, diethyl ether, ethyl acetate, hexane, chloroform and trimesic acid were supplied from Sigma Aldrich and Fluka and were used as received without any further purification unless mentioned.

Melting points were tested on an electrothermal-digital apparatus. nuclear magnetic resonance (NMR) spectra were determined using a Bruker 400 MH_Z_ (for ^1^H) and 100 MHz (for ^13^C) Avance III spectrometer. The infrared spectral data were detected on Bruker alpha FTIR. The elemental analyses of the elements (C, H and N) were carried out on Euro EA elemental analyzer 300.

### 3.1 Preparation of trimesic hydrazide hydrazone **(3a-3m)**

A mixture of 1.0 mmol (0.25 g) of trimesic trihydrazide (**1**) and 3.0 mmol of aromatic aldehydes (**2a-2m**) in 15 mL ethanol was refluxed for 12 h with catalytic amount of glacial acetic acid, Figure 1. The mixture was cooled then the precipitate was collected and recrystallized from DMF/H_2_O [27].

*Tris(thiophen-2-ylmethylene)benzene-1,3,5-tricarbohydrazide* **(3a)**

White powder, yield = 92%, mp: dc > 250 °C, IR spectrum (νcm^−1^): 3236 (NH), 1659 (Amide carbonyl group, C=O), 1512 (C=N). ^1^Н-NMR (d^6^-DMSO), (δ ppm): 7.16–7.18 (t, 3H (J = 4.30 Hz), Aromatic), 7.52–7.53 (d, 3H (J = 2.96H), Aromatic), 7.72–7.73 (d, 3H (J = 5.01Hz), Aromatic), 8.60 (s, 3H, N=CH), 8.70 (s, 3H, Aromatic), 12.16 (s, 3H, NH). ^13^C-NMR(d^6^-DMSO) spectrum, (δ ppm): 127.9, 129.2, 129.7, 131.4, 134.0, 138.7, 143.6, 161.7. Elemental Analysis C_24_H_18_N_6_O_3_S_3_ (534.63) (%) found C 53.06; H 3.73; N 15.46, calculated C 53.92; H 3.39; N 15.72.

*Tris(furan-2-ylmethylene)benzene-1,3,5-tricarbohydrazide* **(3b)**

White powder, yield = 94%, mp: 246–248 °C, IR spectrum (ν cm^−1^): 3205 (NH), 1650 (Amide carbonyl group, C=O), 1513 (C=N). ^1^Н-NMR (d^6^-DMSO) spectrum (δ ppm): 6.67–6.68 (t, 3H(J = 5.04Hz), Aromatic), 6.99–7.00 (d, 3H(J = 3.28Hz), Aromatic), 7.89 (s, 3H, Aromatic), 8.38 (s, 3H, N=CH), 8.62 (s, 3H, Aromatic), 12.1 (s,3H, NH). ^13^C-NMR (d^6^-DMSO) spectrum, (δ ppm): 112.7, 114.6, 130.2, 134.5, 138.6, 145.9, 149.7, 162.6. Elemental Analysis C_24_H_18_N_6_O_6_ (486.44) (%) found C 59.14; H 3.90; N 17.34. calculated C 59.26; H 3.73; N 17.28.

*Tris(4-(dimethylamino)benzylidene)benzene-1,3,5-tricarbohydrazide* **(3c)**

Yellow powder, yield = 91%, mp: dc > 250 °C. IR spectrum (ν cm^−1^): 3212 (NH), 1651 (Amide carbonyl group, C=O), 1589 (C=N). ^1^Н-NMR (d^6^-DMSO) spectrum (δ ppm): 2.99 (s, 18H, N-(CH_3_)_2_), 6.77–6.79 (d, 6H(J = 8.92Hz), Aromatic), 7.57–7.59 (d, 6H(J = 8.85Hz), Aromatic), 8.36 (s, 3H, N=CH), 8.58 (s, 3H, Aromatic), 11.88 (s, 3H, NH). ^13^C-NMR(d^6^-DMSO) spectrum (δ ppm): 39.7, 111.7, 121.3, 128.5, 129.3, 134.3, 149.2, 151.6, 161.1. Elemental Analysis C_36_H_39_N_9_O_3_ (645.75) (%) found C 66.21; H 6.52; N 18.60. calculated C 66.96; H 6.09; N 19.52.

*Tris(4-hydroxy-3,5-dimethoxybenzylidene)benzene-1,3,5-tricarbohydrazide* **(3d)**

Green powder, yield = 89%, mp: dc > 250 °C. IR spectrum (ν cm^−1^): 3394(OH), 3251 (NH), 1650 (Amide carbonyl group, C=O), 1556 (C=N). ^1^Н-NMR (d^6^-DMSO) spectrum (δ ppm): 3.84 (s, 18H, OCH_3_), 7.01 (s, 6H, Aromatic), 8.36 (s, 3H, N=CH), 8.55 (s, 3H, Aromatic), 8.99 (s, 3H, OH),12.08 (s,3H, NH). ^13^C-NMR (d^6^-DMSO) spectrum (δ ppm): 56.0, 104.7, 124.2, 129.6, 134.2, 138.1, 148.1, 149.2,161.9. Elemental Analysis C_36_H_36_N_6_O_12_ (744.7) (%) found C 57.91; H 4.95; N 11.20. calculated C 58.06; H 4.87; N 11.29.

*Tris((E)-3-phenylallylidene)benzene-1,3,5-tricarbohydrazide* **(3e)**

White powder, yield = 93%, mp: 218–220 °C, IR spectrum (ν cm^−1^): 3215 (NH), 1651 (Amide carbonyl group, C=O), 1543 (C=N). ^1^Н-NMR(d^6^-DMSO) spectrum (δ ppm): 7.11–7.13 (d, 6H(J = 6.36Hz), Aromatic), 7.35–7.43 (m, 9H, Aromatic), 7.65–7.67 (d, 6H(J = 7.35Hz), HC = CH), 8.29–8.31 (d, 3H(J = 7.60Hz), N=CH), 8.64 (s, 3H, Aromatic), 12.10 (s, 3H, NH). ^13^C-NMR(d^6^-DMSO) spectrum (δ ppm): 125.5, 127.1, 128.8, 128.9, 129.8, 134.0, 135.8, 139.5, 150.5, 161.78. Elemental Analysis C_36_H_30_N_6_O_3_ (594.66) (%) found C 71.97; H 5.45; N 14.70. calculated C 72.71; H 5.08; N 14.13.

*Tris(2,4,5-trimethoxybenzylidene)benzene-1,3,5-tricarbohydrazide* **(3f)**

White powder, yield = 93%, mp: dc > 250 °C. IR spectrum (ν cm^−1^): 3210 (NH), 1651 (Amide carbonyl group, C=O), 1556 (C=N). ^1^Н-NMR (d^6^-DMSO) spectrum (δ ppm): 3.79 (s, 9H, OCH_3_), 3.87 (s, 9H, OCH_3_), 3.89 (s, 9H, OCH3), 6.72 (s, 3H, Aromatic), 7.31 (s, 3H, Aromatic), 8.61 (s, 3H, N=CH), 8.79 (s, 3H, Aromatic), 12.00 (s, 3H, NH). ^13^C-NMR(d^6^-DMSO) spectrum (δ ppm): 56.2, 56.4, 57.0, 98.3, 108.0, 113.7, 130.0, 134.6, 143.7, 144.4, 152.7, 154.0, 162.1. Elemental Analysis C_39_H_42_N_6_O_12_ (786.78) (%) found C 58.80; H 5.72; N 11. 60. calculated C 59.54; H 5.38; N 10.68.

*Tris(2-chlorobenzylidene)benzene-1,3,5-tricarbohydrazide* **(3g)**

White powder, yield = 88%, mp: dc > 250 °C. IR spectrum (ν cm^−1^): 3215 (NH), 1652 (Amide carbonyl group, C=O), 1558 (C=N). ^1^Н-NMR(d^6^-DMSO) spectrum (δ ppm): 7.47–7.58 (m, 9H, Aromatic), 8.07–8.09 (d, 3H(J = 9.28z), Aromatic), 8.73 (s, 3H, N=CH), 8.94 (s, 3H, Aromatic), 12.40 (s, 3H, NH). ^13^C-NMR (d^6^-DMSO) spectrum (δppm): 126.9, 127.6, 129.9, 130.0, 131.3, 131.7, 133.3, 133.9, 144.5, 161.9. Elemental Analysis C_30_H_21_Cl_3_N_6_O_3_ (619.89) (%) found C 57.87; H 5.61; N 10.83. calculated C, 58.13; H 5.38; N, 10.68.

*Tris(2,5-dimethylbenzylidene)benzene-1,3,5-tricarbohydrazide* **(3h)**

White powder, yield = 85%, mp: dc > 250 °C. IR spectrum) ν cm^−1^ (: 3220 (NH), 1668 (Amide carbonyl group, C=O), 1556 (C=N). ^1^Н-NMR (d^6^-DMSO) spectrum δ ppm (: 2.33 (s, 9H, CH_3_), 2.42 (s, 9H, CH_3_) 7.17 (s, 6H, Aromatic), 7.73 (s, 3H, Aromatic), 8.66 (s, 3H, N=CH), 8.78 (s, 3H, Aromatic), 12.16 (s, 3H, NH). ^13^C-NMR (d^6^-DMSO) spectrum (δ ppm (: 18.1, 24.3, 126.5, 128.5, 129.1, 129.4, 131.4, 134.7, 136.7, 137.8, 143.0, 162.3. Elemental Analysis C_36_H_36_N_6_O_3_ (600.71) (%) found C 71.52; H 5.95; N 14.14. calculated C,71.98; H,6.04; N,13.99.

*Tris(4-methoxy-2,5-dimethylbenzylidene)benzene-1,3,5-tricarbohydrazide* **(3i)**

White powder, yield = 91%, mp: dc > 250 °C. IR spectrum (νcm^−1^): 3187 (NH), 1660 (Amide carbonyl group, C=O), 1591 (C=N). ^1^Н-NMR (d^6^-DMSO) spectrum (δ ppm): 2.16 (s, 9H, CH_3_), 2.43 (s, 9H, CH_3_),3.83 (s, 9H, OCH_3_), 6.84 (s, 3H, Aromatic), 7.70 (s, 3H, Aromatic), 8.60 (s, 3H, N=CH), 8.72 (s, 3H, Aromatic),12.03 (s, 3H, NH). ^13^C-NMR(d^6^-DMSO) spectrum (δ ppm): 16.0, 19.2, 55.8, 112.8, 124.1, 128.2, 129.9, 131.1, 134.7, 137.2, 147.5, 159.2, 162.1. Elemental Analysis C_39_H_42_N_6_O_6_ (690.79) (%) found C 66.95; H 6.78; N 12.79. calculated C 67.81; H 6.13; N 12.17.

*Tris(4-cyanobenzylidene)benzene-1,3,5-tricarbohydrazide* **(3j)**

Yellow powder, yield = 90%, mp: dc > 250 °C. IR spectrum (νcm^−1^): 3210 (NH), 2360 (C≡N), 1665 (Amide carbonyl group, C=O), 1555 (C=N). ^1^Н-NMR(d^6^-DMSO) spectrum (δ ppm): 7.96 (s, 12H, Aromatic), 8.56 (s, 3H, N=CH), 8.70 (s, 3H, Aromatic), 12.47 (s, 3H, NH). ^13^C-NMR(d^6^-DMSO) spectrum (δ ppm): 112.0, 118.6, 127.7, 130.2, 132.7 133.8, 138.5, 146.6, 162.2. Elemental Analysis C_33_H_21_N_9_O_3_ (591.58) (%) found C 65.98; H 4.01; N 20.78. calculated C 67.00; H 3.58; N 21.31.

*Tris(naphthalen-1-ylmethylene)benzene-1,3,5-tricarbohydrazide* **(3k)**

Yellow powder, yield = 88%, mp: dc > 250 °C. IR spectrum (ν cm^−1^): 3187 (NH), 1658 (Amide carbonyl group, C=O), 1578 (C=N). ^1^Н-NMR(d^6^-DMSO) spectrum (δ ppm): 7.63–7.72 (m, 12H, Aromatic), 8.01–8.09 (m, 6H, Aromatic), 8.80 (s, 3H, N=CH), 8.87–8.89 (d, 3H(J = 8.60Hz), Aromatic), 9.22 (s, 3H, Aromatic), 12.36 (s, 3H, NH). ^13^C-NMR(d^6^-DMSO) spectrum (δ ppm): 126.1, 126.8, 127.7, 127.9 128.3, 129.3, 129.8 130.3, 130.8, 130.7, 131.2, 134.7, 148.8, 162.4. Elemental Analysis C_42_H_30_N_6_O_3_ (666.73) (%) found C 75.23; H 4.31; N 12.44. calculated C 75.66; H 4.54; N, 12.60.

*Tris(quinolin-4-ylmethylene)benzene-1,3,5-tricarbohydrazide* **(3l)**

White powder, yield = 87%, mp: 239–241 °C. IR spectrum (ν cm^−1^): 3234 (NH), 1671 (Amide carbonyl group, C=O), 1559 (C=N). ^1^Н-NMR(d^6^-DMSO) spectrum (δ ppm): 7.77–7.81 (t, 3H(J = 7.31Hz), Aromatic), 7.85–7.89 (t, 3H(J = 7.45Hz), Aromatic),7.92–7.93 (d, 3H(J = 3.92Hz), Aromatic), 8.13–8.15 (d, 3H(J = 8.2Hz), Aromatic), 8.79–8.77 (d, 3H(J = 8.36Hz), Aromatic), 8.84 (s, 3H, N=CH), 9.03–9.04 (d, 3H(J = 3.99Hz), Aromatic), 9.21 (s, 3H, Aromatic), 12.64 (s, 3H, NH). ^13^C-NMR (d^6^-DMSO) spectrum (δ ppm): 120.5, 125.1, 128.2, 130.2, 130.3,130.6, 130.8, 134.5, 137.6, 146.3, 148.9, 150.9, 162.6. Elemental Analysis C_39_H_27_N_9_O_3_ (669.69) (%) found C 69.84; H 3.97; N 18.73. calculated C 69.95; H 4.06; N 18.82.

*Tris((2-hydroxynaphthalen-1-yl)methylene)benzene-1,3,5-tricarbohydrazide***(3m)** [31]

Yellow powder, yield = 89%, mp: 245–246 °C, IR spectrum (ν cm^−1^): 3441 (OH), 3213 (NH), 1649 (Amide carbonyl group, C=O), 1573 (C=N). ^1^Н-NMR (d^6^-DMSO) spectrum (δ ppm): 7.26–7.29 (d, 3H(J = 8.96Hz), Aromatic), 7.42–7.45 (t, 3H(J = 7.34Hz), Aromatic), 7.63–7.65 (t, 3H(J = 7.64Hz), Aromatic), 7.92–7.94 (d, 3H(J = 7.92Hz), Aromatic), 7.97–7.99 (d, 3H(J = 8.96Hz), Aromatic), 8.32–8.34 (d, 3H(J = 6.17Hz), Aromatic), 8.87 (s, 3H, N=CH), 9.59 (s, 3H, Aromatic), 12.64 (s, 3H, OH), 12.72 (s, 3H, NH). ^13^C-NMR(d^6^-DMSO) spectrum (δ ppm): 108.5, 118.8, 120.8, 123.6, 127.8, 128.9, 129.9, 131.6, 133.0, 133.7, 147.6, 158.1, 161.2, 162.2. Elemental Analysis C_42_H_30_N_6_O_6_ (714.72) (%) found C 70.34; H 4.48; N 12.02. calculated C 70.58; H 4.23; N 11.76.

### 3.2 Biological properties

#### 3.2.1 Antimicrobial activity

Bacterial isolates used in this study were obtained from the Central Laboratories, Jordan Ministry of Health. The clinical isolates *(Escherichia coli*; *Klebsiella pneumoniae*; *Proteus mirabilis*; *Salmonella enteritidis, Pseudomonas aeruginosa*; *Staphylococcus aureus; Enterococcus faecalis*; *Bacillus cereus*) were grown in Muller Hinton Broth media for 24 h at 37 °C. The biological activity of the compounds was determined by establishing the minimum inhibitory concentration (MIC, mg/μL) using the micro-broth dilution method as described by Hannan [32]. Stock solutions of the compounds in DMSO were prepared according to CLSI guidelines [33]. The in vitro MIC was carried out in standard sterile 96 well flat bottom micro-titer plates. The layout was designed such that each row covered a range of concentrations from 500 to 0.5 mg/μL of the respective compounds under investigation with positive and negative control well. To each well, 40 mL of the selected compounds at the correct concentration was added and the control well was loaded with a 40 μL of DMSO solvent. Each well then received 150 μL of Muller Hinton media and 10 μL of the bacterial culture that was standardized with 0.5 McFarland turbidity standards. The final concentration of bacteria in the inoculum was approximately 5.0 × 10^7^ CFU/μL Plates were sealed and incubated at 37 °C under atmospheric conditions for 24 h. Micro-titer plates were read using an ELIZA UV–vis spectrometer. The minimal concentration that had an optical density below that of the control was defined as the MIC.

#### 3.2.2 Antioxidant activity assays

The total antioxidant capacity of the tested hydrazones was measured spectrophotometrically by using the s t able 1,1- diphenyl-2-picrul-hydrazilor, 2,2′-azinobis-(3- ethylbenzothiazoline-6-sulfononic acid) free radicals in vitro assays. DPPH and ABTS have different mechanisms of neutralizing their free radical character. DPPH^•^ absorbance decreases in presence of hydrogen-donating antioxidants due to the formation of the stable DPPH-H compound while ABTS is involved in an electron transfer process. Percent inhibition was calculated using Eq. (1):

(1) $$\text{\% ​Inhibition}=\frac{\text{Absorbance ​of ​the ​control}-\text{absorbance ​of ​the ​tested ​sample ​}}{\text{Absorbance ​of ​the ​control}}$$

Then, the inhibition concentration 50 (IC_50_) value was calculated based on a linear regression Eq. (2)

(2) (y = ax + b)

from the curve by plotting the Ln concentration on x-axis and percentage of inhibition on y-axis.

#### 3.2.3 DPPH scavenging activity assay

All synthesized compounds were screened for radical scavenging ability against DPPH (2,2-diphenyl-1-picrylhydrazyl) radical. A 1.0 mL of each test compound at different concentrations of (0.005, 0.01, 0.05, 0.10, 0.5 mg/mL) prepared in DMF was mixed with 1.0 mL of recently prepared solution of 0.10 mM DPPH in ethanol. The mixtures were stored in the dark for 30 min, and then, the absorbance of these solutions was obtained at (λ = 517 nm) using DMF solvent as a blank. Radical scavenging activity was determined according to the method of Blois [34]. The chemical response was compared with the one obtained under identical experimental conditions with ascorbic acid.

#### 3.2.4 ABTS^•+^ scavenging activity assay

The total antioxidant capacity (TAC) in the ABTS assay was determined by the published methods [35, 36]. The ABTS assay utilizes the free mono-cation radical of 2′2-azino-bis(3-ethylbenzothiazoline-6-sulphonic acid), generated by the oxidation of ABTS with potassium persulfate. The blue-green colored solution was dark stored for 1 day. The **ABTS**^**•+**^ cation radical solution was prepared by reacting of equivalent quantities of 7 mM of **ABTS**^**•+**^ and 2.4 mM of potassium persulfate (K_2_S_2_O_8_) solution for 16 h at RT in the dark. Before using this solution, it was diluted with methanol to get an absorbance of 0.75 ± 0.02 at 734 nm. The reaction mixture was prepared by mixing 3.0 mL of **ABTS**^**•+**^ solution and 2.0 mL of tested compounds 3a-3 m at various concentrations (0.005, 0.01, 0.05, 0.10) mg/mL. The discoloration of the pre-generated **ABTS**^**•+**^ radical was measured at 734 nm and the chemical response was compared with the one obtained under identical experimental conditions with ascorbic acid.

### 3.3 Absorption and fluorescence spectroscopy

Absorption spectra of all compounds were recorded in DMSO using a Shimadzu UV-Vis absorption spectrometer Model UV 1800 in the spectral range 200–900 nm. The fluorescence spectra were recorded with an Edinburgh instruments fluorometer Model-FS 900SDT in the range 200–900 nm at room temperature. The photophysical studies of all compounds were performed in DMSO solutions, and the basic photophysical characteristics such as the absorption maxima (λ_abs_) and emission maxima (λ_em_) were determined.

### 3.4 Computational method

All electronic structure calculations were performed using the Wavefunction Spartan’18 Parallel Suite [37]. The structures of all prepared derivatives were fully optimized in the gas phase without any symmetry or geometry constraints at the B3LYP level of theory with the polarized 6-31G(d) basis set [38, 39, 40, 41, 42]. The lack of imaginary frequencies in the vibrational mode calculation verified that structures were indeed true minima at the performed level of theory for each derivative.

## 4 Conclusions

Condensation of trimesic trihydrazide and aromatic aldehydes resulted in the formation of new series of hydrazone compounds. Spectral and elemental analysis were used to determine the structures of the synthesized hydrazone compounds. The optimized ground-state geometries of all prepared derivatives were elucidated using DFT calculations at the B3LYP/6-31G(d) level of theory. The results indicate that all structures feature trimeric propeller-shaped arrangements around a central 1,3,5-trisubstituted benzene. Their potential as antioxidants was studied using the **DPPH** and **ABTS**^•+^ scavenging activities. The **3g**, **3i** compounds showed highest DPPH radical scavenging ability. while, **3c**,**3l**, and **3m** were inactive toward DPPH assay. All test compounds exhibited good antioxidant capacity in the ABTS assay. Moreover, the inhibition activity of all hydrazones was found to be concentration dependent. Antimicrobial activity of the derivatives was estimated using a micro-broth dilution method. The MIC of all tested compounds were above 500 μg/mL and considered inactive.

## Declarations

### Author contribution statement

Ibrahim Mhaidat; Ayman Shdefat: Conceived and designed the experiments; Performed the experiments; Analyzed and interpreted the data; Contributed reagents, materials, analysis tools or data; Wrote the paper.

Fadel Alwedian; Taher Ababneh; Hasan Tashtoush: Analyzed and interpreted the data; Contributed reagents, materials, analysis tools or data; Wrote the paper.

### Funding statement

This work was supported by the deanship of Scientific Research and Graduate Studies at 10.13039/501100006418Yarmouk University for providing the financial support of this work [project number 22/2016].

### Data availability statement

The authors do not have permission to share data.

### Declaration of interests statement

The authors declare no conflict of interest.

### Additional information

No additional information is available for this paper.
